# Rapidly Progressing Prostate Cancer With Low Prostate-Specific Antigen and Gleason Score 5+5: A Case Report

**DOI:** 10.7759/cureus.80808

**Published:** 2025-03-19

**Authors:** Kosuke Uchida, Akira Fujisaki, Shin Imai, Yoshiro Otsuki, Tatsuaki Yoneda

**Affiliations:** 1 Department of Urology, Seirei Hamamatsu General Hospital, Hamamatsu, JPN; 2 Department of Pathology, Seirei Hamamatsu General Hospital, Hamamatsu, JPN

**Keywords:** androgen deprivation therapy, gleason score, prostate cancer, prostate-specific antigen, testosterone

## Abstract

An 87-year-old man presented with worsening urinary dysfunction with urinary retention. The prostate-specific antigen was 1.23 ng/mL. Prostate biopsy confirmed adenocarcinoma with a Gleason score of 5+5; the final diagnosis was T4N1M0. Androgen deprivation therapy and immediate whole-pelvic radiotherapy (80 Gy) were administered. Nadir prostate-specific antigen of 0.40 ng/mL two months later increased to 35.92 ng/mL as the disease metastasized to the corpus cavernosum penis, causing malignant priapism. The best supportive care was provided, and the patient died 11 months later. High-grade prostate cancers with low prostate-specific antigen levels are rare but highly aggressive. Androgen deprivation therapy and radiotherapy alone may be insufficient in such cases, highlighting the need for more effective treatment strategies.

## Introduction

Prostate-specific antigen (PSA) is useful for screening prostate cancer, but some cases are diagnosed despite having a low PSA level. Prostate biopsy conducted with PSA levels <4 ng/mL revealed prostate cancer in 15.2% of cases, most of which were insignificant and had a Gleason score (GS) ≤6 [1]. Prostate cancers with a low PSA level and GS 5+5 are rare and account for only 0.04% of the cases [2,3]. High-grade cancer with a low PSA level is biologically aggressive and has a poor prognosis [4-6]. Non-neurogenic dedifferentiation in some high-grade tumors leads to reduced PSA expression and resistance to androgen deprivation therapy (ADT), increasing their malignancy [7]. Furthermore, GS 9-10 prostate cancer with a low PSA has worse outcomes compared with cases of higher PSA, suggesting alternative molecular mechanisms leading to its increased malignancy [4,6,8]. Malignant priapism is a rare condition caused by metastatic cancer, most commonly from bladder or rectal tumors. Although prostate cancer can also lead to malignant priapism, its reported cases are uncommon [9]. Herein, we report a case of a patient previously treated for benign prostatic hyperplasia (BPH) and was diagnosed with GS 5+5 prostate cancer with low PSA levels, which rapidly progressed and was accompanied by malignant priapism, leading to death 11 months after diagnosis.

## Case presentation

**Figure 1:**
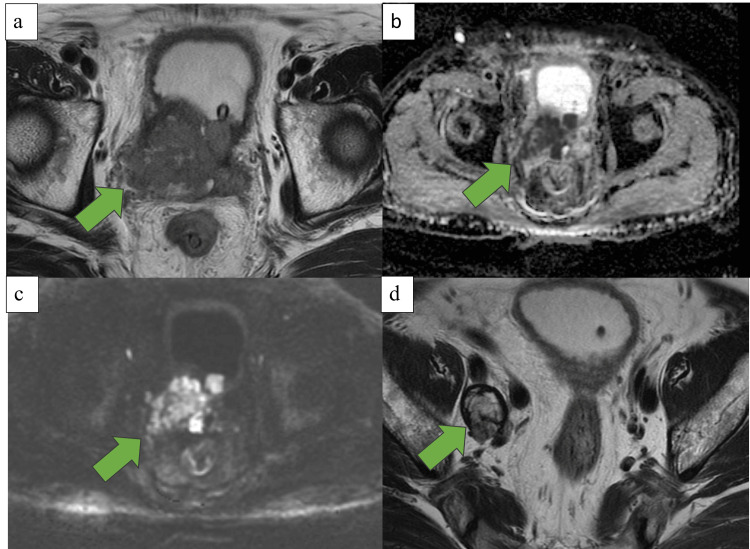
Magnetic resonance imaging a, b, c: T2WI and apparent diffusion coefficient and diffusion-weighted imaging showing prostatic enlargement with a mixed signal mass of 6.0 cm × 5.2 cm × 6.4 cm and prostate cancer with bladder invasion. d: T2WI showing right obturator lymph node enlargement with a mixed signal mass of 2.5 cm × 3.0 cm × 2.7 cm. T2WI, T2-weighted imaging.

An 87-year-old man was diagnosed with BPH without biopsy three years ago, and an alpha-blocker was prescribed. At the first visit, the patient exhibited an Eastern Cooperative Oncology Group performance status of 0; there was no family history; hypertension and hyperlipidemia were present; and PSA was 1.96 ng/mL (normal value: 0-4 ng/mL). Prostate volume was 32 mL, and digital rectal examination (DRE) showed no induration suggestive of malignancy. One year prior, the patient presented with asymptomatic gross hematuria. At that time, transabdominal echo and non-contrast computed tomography (CT) revealed no cause of hematuria. Urine cytology was negative, and the PSA was 1.68 ng/mL. DRE was not performed. Cystoscopy revealed oozing from the prostatic urethra, and the patient was diagnosed with bleeding from BPH. Thereafter, microscopic hematuria was observed; however, gross hematuria was not. The urinary dysfunction had worsened one month ago, resulting in urinary retention, prompting the patient to visit the emergency department. A urinary catheter was inserted, and surgery for BPH was planned. Magnetic resonance imaging (MRI) performed preoperatively revealed prostate cancer with bladder invasion and pelvic lymph node metastasis (Figure 1).

**Figure 2:**
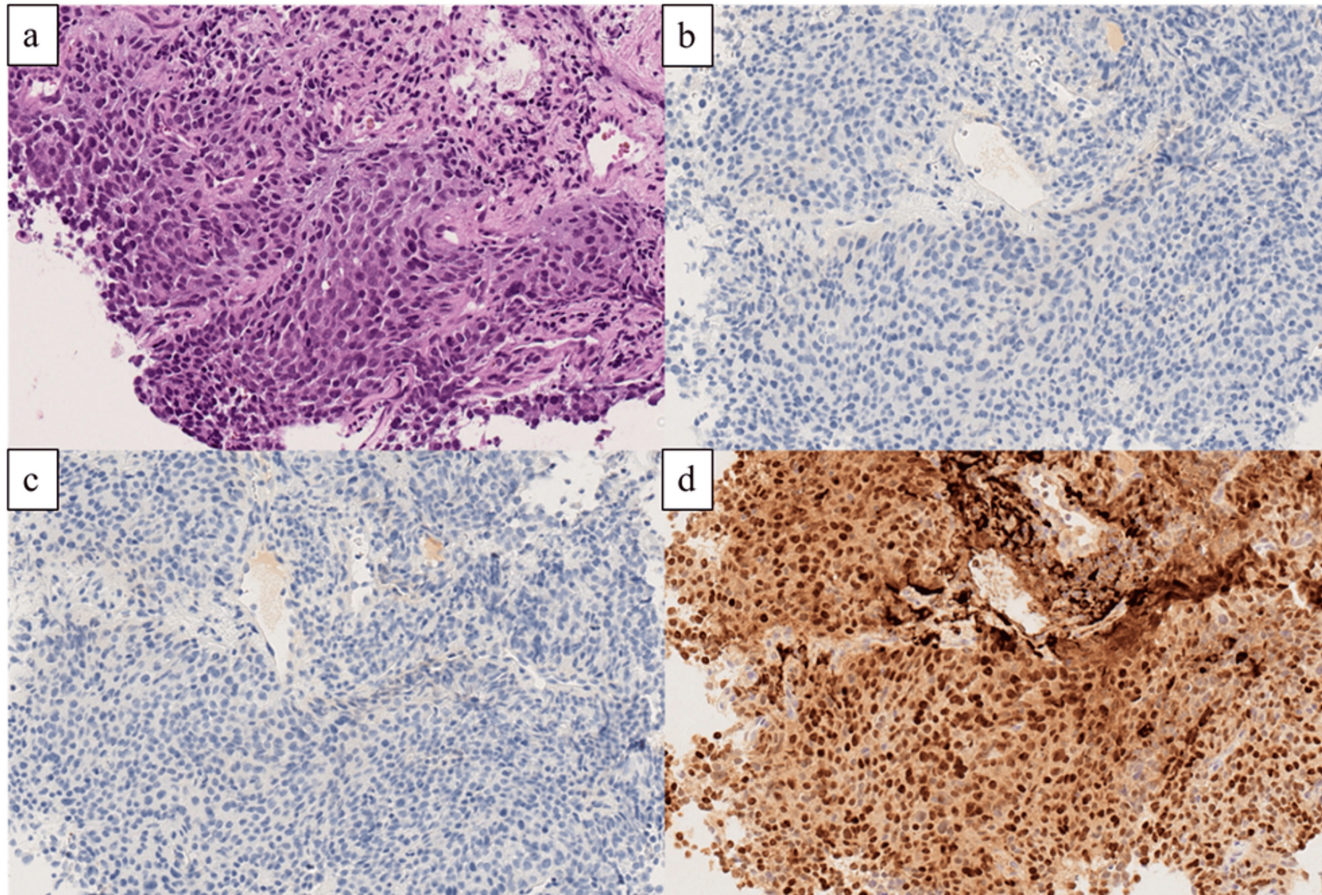
Pathology Microscopic findings of the prostate biopsy specimen show poorly differentiated adenocarcinoma, with a Gleason score 5+5=10 (hematoxylin and eosin staining, ×20) (a). Immunohistochemistry results show tumor cells negative for synaptophysin (×20) (b), chromogranin A (×20) (c), and positive for NKX3.1 (×20) (d).

A stony-hard mass was palpable on DRE. Tumor markers were normal: PSA 1.23 ng/mL, neuron-specific enolase 11.4 ng/mL (normal value: 0-16.3 ng/mL), and pro-gastrin-releasing peptide 42.87 pg/mL (normal value: 0-81.0 pg/mL). A prostate biopsy showed prostate cancer with a GS of 5+5. Neuroendocrine differentiation or small cell carcinoma was not detected (Figure 2).

Contrast-enhanced CT and bone scintigraphy revealed no evidence of distant metastases, and T4N1M0 prostate cancer was diagnosed.

**Figure 3:**
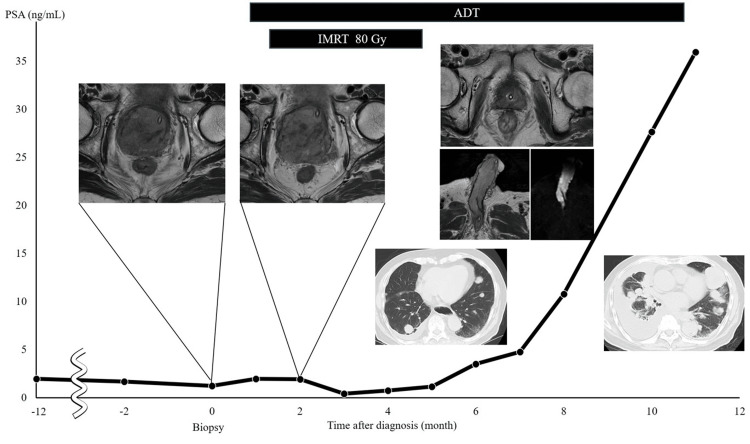
Changes in the tumor markers and imaging findings from the first visit PSA, prostate-specific antigen; ADT, androgen deprivation therapy; IMRT, intensity-modulated radiation therapy.

ADT monotherapy using a luteinizing hormone-releasing hormone antagonist and immediate whole-pelvic radiotherapy (RT) (80 Gy in 2.0 Gy per fraction) were administered. Two months after treatment, the PSA nadir was 0.40 ng/mL and MRI revealed marked shrinkage of the prostate (Figure 3); subsequently, the PSA increased. Four months later, the PSA increased to 1.14 ng/mL, and the patient developed priapism. MRI revealed a metastatic tumor in the corpus cavernosum of the penis, leading to a diagnosis of malignant priapism due to metastasis to the corpus of cavernosum of the penis from prostate cancer. CT revealed metastases to the para-aortic lymph nodes, lungs, and adrenal glands. Because of the patient’s poor general condition, supportive care was selected instead of treatment for castration-resistant prostate cancer (CRPC), such as androgen receptor signaling inhibitors (ARSI). PSA increased to 35.92 ng/mL, and the patient died of cancer 11 months after the initial diagnosis (Figure 3).

## Discussion

Prostate cancer can be present even if the PSA level is low and DRE is normal. In a study of 2,950 men with PSA <4 ng/mL, 449 (15.2%) were diagnosed with prostate cancer, and 361 (80%) had insignificant cancer [1]. However, GS 5+5 prostate cancer with a low PSA is extremely rare. Among 498,067 prostate cancers, only 221 (0.04%) had PSA <4 ng/mL and GS 5+5 [2], and another study of 99,620 cases found 80 (0.04%) had PSA <3 ng/mL and GS 5+5 [3]. In cases of prostate cancer with low PSA levels, neuroendocrine differentiation or small cell carcinoma should be considered in the differential diagnosis [8,10]. De novo neuroendocrine differentiation is rarely encountered; therefore, immunohistochemical evaluation of PSA, NKX3.1, chromogranin, and synaptophysin is required [7].

High-grade prostate cancer with low PSA is difficult to diagnose. Unlike typical cases diagnosed based on elevated PSA, diagnosis of high-grade prostate cancer often depends on clinical symptoms and DRE. In this case, preoperative MRI for BPH screening incidentally detected prostate cancer. Although routine MRI screening for prostate cancer is not recommended due to cost-benefit considerations, it has been recognized as useful for evaluating prostate transition zone and morphology before BPH surgery [11].

High-grade prostate cancer with low PSA levels is associated with poor prognosis, as reported in multiple studies [4,6]​. One study found that patients with PSA <4.0 ng/mL showed significantly worse prostate cancer-specific mortality (PCSM) than those with PSA of 4.0-10.0 ng/mL, particularly in patients with GS 9-10 prostate cancer [4]. Furthermore, the relationship between PSA and the prognosis of high-grade prostate cancer follows a nonlinear pattern. Studies have shown a J-shaped association, where patients with very low PSA (≤2.5 ng/mL) have nearly double the PCSM risk compared with those with a PSA of 4.1-10.0 ng/mL [6].

Patients with PSA ≤2.5 ng/mL exhibited poor outcomes even after RT, suggesting that additional therapeutic interventions might be necessary [10]. Those with PSA ≤5 ng/mL undergoing RT had significantly higher PCSM than those with PSA of 5.1-10 ng/mL [6]. Findings in a study on non-metastatic GS 8-10 prostate cancer revealed that PSA <4.0 ng/mL was linked to worse PCSM following RT compared with PSA of 4.0-10.0 ng/mL [12].

Given the unique molecular profile of GS 9-10 prostate cancers, standard ADT is often insufficient. Some tumors manifest low AR activity or neuroendocrine differentiation, leading to poor response to ADT [7,13]. These tumors frequently harbor genomic alterations in TP53, PTEN, and RB1, common in metastatic CRPC, contributing to resistance to ADT [14]. Low PSA production in patients with high-grade prostate cancer is linked to suppressed AR signaling, with TP53 and RB1 mutations promoting neuroendocrine features [15] and PTEN loss activating the PI3K/AKT pathway [16]. These molecular changes highlight the need for alternative therapeutic strategies beyond ADT alone.

Consequently, ADT monotherapy may be inadequate; additional systemic therapies, such as ARSI or docetaxel, should be considered [17]. Although ARSI is not covered by insurance for non-metastatic prostate cancer, studies suggest that ADT alone may be insufficient and ARSI or docetaxel could be beneficial. A recent meta-analysis has suggested that adding docetaxel to ADT and radiation significantly reduces PCSM in patients with high-grade, non-metastatic, treatment-naïve prostate cancer with low PSA levels, particularly in those with good performance status [18]. In this case, given the patient’s advanced age (87 years) at diagnosis, treatment was limited to ADT monotherapy and RT. The prognosis in our case was extremely poor, with survival lasting only 11 months from diagnosis.

Advanced prostate cancer often manifests with distant metastases; in rare cases, these can involve atypical sites such as the corpus cavernosum penis. Malignant priapism, though uncommon, is an important clinical manifestation of metastatic prostate cancer. In this case, priapism developed following prostate irradiation, despite MRI findings indicating prostate shrinkage, suggesting hematogenous or retrograde venous dissemination as a potential mechanism of metastasis. Priapism occurred due to metastasis to the corpus cavernosum penis. According to previous reports, 512 cases of metastases to the corpus cavernosum from malignant tumors have been documented, with the most common primary sites of malignancy being the bladder (30.6%) and prostate (29.6%) [9]. Although penile metastases from prostate cancer are extremely rare, their clinical significance is substantial, as they are often associated with advanced disease and poor prognosis. The proposed mechanisms of metastasis include hematogenous spread, retrograde venous dissemination via the Santorini plexus, lymphatic spread, and direct extension from adjacent organs [19]. Given that penile metastases typically reflect advanced-stage disease, treatment is primarily palliative, aiming to alleviate symptoms and improve quality of life. Surgical intervention, such as partial or total penectomy, is considered in patients with severe refractory symptoms, while pain management strategies, including nerve blocks and analgesics, are tailored to individual needs [19]. In the present case, given the patient's advanced age and disease progression, a palliative approach focusing on pain control was chosen as the primary management strategy.

## Conclusions

Prostate cancer with low PSA levels and GS 10 is biologically aggressive and exhibits rapid progression. Treatment with ADT for such tumors is ineffective; the prognosis is extremely poor. ADT and RT alone may be insufficient, necessitating a multimodal treatment approach. Given the poor prognosis of these tumors, more effective therapeutic strategies should be considered.
